# "Familial" versus "sporadic" intellectual disability: contribution of subtelomeric rearrangements

**DOI:** 10.1186/1755-8166-5-4

**Published:** 2012-01-19

**Authors:** Maryam Rafati, Mohammad R Ghadirzadeh, Yaser Heshmati, Homeira Adibi, Zarrintaj Keihanidoust, Mohammad R Eshraghian, Jila Dastan, Azadeh Hoseini, Marzieh Purhoseini, Saeed R Ghaffari

**Affiliations:** 1 Department of Medical Genetics, Tehran University of Medical Sciences, Tehran, Iran; 2 Comprehensive Genetic Center, Hope Generation Foundation, Tehran, Iran; 3 Tehran Welfare Organization, Tehran, Iran; 4 Division of Pediatric Neurology, Department of Pediatrics, Tehran University of Medical Sciences, Tehran, Iran; 5 Department of Epidemiology and Biostatistics, School of Public Health, Tehran University of Medical Sciences, Tehran, Iran; 6 Gene Clinic, Tehran, Iran

**Keywords:** Familial Intellectual Disability, Mental Retardation, Subtelomeric Rearrangements, Family History

## Abstract

**Background:**

Cryptic subtelomeric rearrangements have been proposed as a significant cause of sporadic intellectual disability (ID) but the role of such aberrations in familial ID has not yet been studied. As positive family history of ID had been proposed as an important and significant predicting factor of subtelomeric rearrangements, it was assumed that the contribution of subtelomeric aberrations in familial ID would be much more than the sporadic ones. Three hundred and twenty two patients from 102 unrelated families with more than two ID patients in the first degree relatives have been investigated. Assessment of subtelomeric rearrangements were carried out using Multiplex Ligation-Dependent Probe Amplification (MLPA) technique. Detected aberrations were then confirmed by Fluorescence in Situ Hybridization (FISH) method.

**Results:**

Among the families studied, 27.4% had 4-12, 36.3% had 3 and 36.3% had 2 affected individuals in the first degree relatives. One unbalanced translocation and 4 polymorphic changes were detected. The prevalence of clinically significant subtelomeric rearrangements was 0.98%.

**Conclusion:**

This is the first investigation of subtelomeric aberrations in a large sample set of familial ID patients. Our results show that the contribution of subtelomeric rearrangements to familial ID is not as much as what had been determined for sporadic ones in the literature. Moreover, this study shows that the positive family history by alone, cannot be the most important and determining indicator of subtelomeric aberrations while it would be a good predicting factor when associated with dysmorphism or congenital malformations. These findings propose that other cryptic chromosomal abnormalities or even single gene disorders may be the main cause of familial ID rather than subtelomeric aberrations.

## Background

Intellectual Disability, formerly Mental Retardation, is a lifelong disability with the prevalence of 1-3% that imposes a heavy burden on the society, health care system and affected families. It is defined as having the following components: 1) significantly abnormal intellectual performance determined by IQ tests; 2) onset before the age of 18; 3) impairment of the adaptation to the environment [1]. While the causes of sporadic Intellectual disability (ID) have been thoroughly investigated during recent decades and some new genes causing autosomal recessive ID have been recently reported, [2], still little is known about the underlying genetic causes of familial ID especially in non-recessive types of ID. This could be attributed to the low incidence of familial ID in western countries, where most of the studies have been carried out. Sporadic ID is caused by extremely heterogeneous factors including environmental, chromosomal and monogenic factors. It is estimated that half of all cases of sporadic ID is caused by genetic factors [3], however, the contribution of these causes is much higher to the familial ones. Assessment of the genetic basis of familial ID can therefore lead to the prevention of the recurrence of ID in these families as well as elucidating the new underlying genetic causes of ID.

In 1995, Flint et al proposed subtelomeric rearrangements as a significant cause of ID [4]. Many studies have been carried out afterward, to identify the prevalence and genotype phenotype correlation of subtelomeric rearrangements in these patients [5-10].

The prevalence of subtelomeric aberrations has been reported widely variable among different studies depending on the inclusion criteria selected. It is reported from as low as zero in unselected mildly affected cases [11], to 9-15% in highly selected moderate to severe mental retarded patients with dysmorphic features, congenital malformations and the family history of abortions or previous affected cases [6,7,12]. Based on a retrospective evaluation of the mentioned common clinical features in the patients with known subtelomeric aberrations, a checklist for preselecting of cases to improve the rate of informative tests has been developed [13]. This checklist and some other investigations suggested prenatal onset growth retardation and positive family history of ID, as important indicators of subtelomeric rearrangements. Regarding the proposed predicting power of positive family history, it was assumed that investigation of subtelomeric aberrations could have a higher diagnostic yield in familial rather than sporadic ID. Due to the small family sizes of the previous studies and the limited number of familial ID patients, to the best of our knowledge, no report on a large sample set of familial ID has so far been published to evaluate the contribution of subtelomeric rearrangements in familial ID.

Here, we report the results of the assessment of subtelomeric rearrangements in 322 affected individuals from 102 families with recurrent ID in the first degree relatives, among families registered in Tehran Welfare Organization. Moreover, an analytical overview comparing the results of this study to the main surveys of subtelomeric rearrangements (mainly in sporadic ID patients), with specific attention to the sample set features and proportion of hereditary subtelomeric changes, is also presented.

## Results

Three hundred and twenty two patients from 102 unrelated Iranian families, including 290 affected individuals in sibships and 32 affected parents were included in this study. In 17 families the fathers, in 3 the mothers and in 6, both of the parents were mentally retarded. Overall, there were 182 (56.5%) male and 140 (43.5%) female individuals among these patients. After excluding the affected parents, the number and percentage of the male and female patients changed to 159 (54.8%) and 131 (45.2%) respectively. The mean age of the patients was 26.4 years.

The number of affected individuals with ID in the first degree relatives of each family ranged from 2 to 12. Among the families studied, 28 (27.4%) had 4 or more affected individuals in the first degree relatives. The number of affected members in the mentioned families was 12 in 1 (0.98%) family, 8 in 2 (2%) families, 6 in 2 (2%) families, 5 in 5 (5%) families and 4 in 18 (17.5%) families. In the remaining 74 families, 37 (36.3%) had 3 and 37 (36.3%) had 2 affected individuals in the first degree relatives. The mean number of affected individuals in the first degree relatives of the studied families was 3.16. There were 7 families having affected individuals in all generations including the first, second and third degree relatives.

Pedigree analysis was carried out on 322 patients from 102 families with familial ID. As it is generally accepted that all of the affected members in each individual family harbor the same mutation, one patient from each family was selected for assessment of subtelomeric rearrangements based on the availability of the patients and parents and/or patients preferences. In case of subtelomeric aberrations detected, investigation on other family members including the parents, other affected individuals and the normal siblings was carried out to examine the clinical significance of that finding.

Five subtelomeric rearrangements were detected by MLPA technique which could be subdivided into two groups. The first group included a hereditary subtelomeric aberration in which MLPA showed a partial gain (trisomy) of 9pter and a partial loss (monosomy) of 13qter in the patient using the P036 and P070 probemixes. The patient was an 11-year old girl with dysmorphic features, severe ID, visual impairment and behavioral disorder. Metaphase FISH was carried out in the patient and her parents, both to confirm the MLPA finding and to delineate the origin of this rearrangement. It showed that the father carried a balanced translocation between the telomeric regions of the short arm of chromosome 9 and the long arm of chromosome13.

The second group consisted of rearrangements which were firstly found in the affected individuals and further investigations showed the same finding in the other normal family members. These included 4qter gains in 3 families and 3pter losses in one family.

## Discussion

Three hundred and twenty two patients from 102 families with recurrent ID were investigated in this study, among which, one pathogenic change and 4 polymorphic changes from two types of previously reported familial variations were detected [14].

Demographic data showed that there was no notable difference between the proportion of males (56.4%) and females (43.6%) in the studied population due to the exclusion of Fragile-X syndrome as the most common cause of X-linked intellectual disability. Due to an excess of the number of affected fathers (23 affected fathers in comparison with 9 affected mothers), the percentage of males and females changed to 54.8% and 45.2% respectively when the affected parents were excluded.

The only pathogenic subtelomeric aberration detected, was a partial trisomy of chromosome 9p telomeric region and partial monosomy of the terminal region of the long arm of chromosome 13 (13qter) in an affected individual inherited from a clinically normal balanced carrier father. There were one spontaneous abortion and three affected children with multiple congenital anomalies, dysmorphism and severe mental retardation in the family. The prevalence of clinically significant subtelomeric rearrangements in this study was therefore 0.98% which is lower than most of the previous studies.

The sample set of the present study is different from all of the previous investigations, as it is confined to just familial ID cases. Based on the mentioned feature, de novo subtelomeric rearrangements were not expected to be detected among the studied population and therefore the obtained results should be compared to the frequency of hereditary and not the overall subtelomeric aberrations reported in other investigations.

An analytical overview of 21 main surveys of subtelomeric rearrangements with specific attention to the sample set features and proportion of hereditary subtelomeric changes, is presented in table 1.

**Table 1:** The analytical overview of 21 subtelomeric screening studies focusing on sample set features and proportion of hereditary subtelomeric rearrangements

Reference	Number of Patients studied	Method of Analysis	Number of Families with "Familial ID"	Patients Selection	Number of Subtelomeric Rearrangements			Frequency of Subtelomeric Rearrangements	
					
					Overall	De novo	Hereditary	Overall	Hereditary
Knight et al [1999]	466 Mild ID: 182 Moderate to severe ID: 284	FISH	9 Reported	Selected (high proportion of moderate to severe ID)	22	12	10	Mild: 0.5% Mod-severe: 7.4%	Mild: 0% Mod-severe: 3.52%

Ballif et al [2000]	154	FISH	Not Reported	No selection	4	4	0	2.7%	0

Fan et al [2001]	150	FISH	Not Reported	Selected (dysmorphic features +/- congenital malformations)	6	2	4*^a^*	4%	2.7%

Riegel et al [2001]	254	FISH	10*^b^*	Highly selected (dysmorphic features +/- multiple congenital anomalies +/- positive family history)	13	7	6	5%	2%

Rosenberg et al [2001]	120	Microsatellite Marker	Familial cases are excluded	Highly selected	5	1	4	4.1%	3.3%

Rossi et al [2001]	200	FISH	53*^b^*	Highly selected (dysmorphic features +/- major malformations +/- positive family history)	13	7	6*^c^*	6.5%	3%

Anderlid et al [2002]	111	FISH	40*^b^*	Highly selected (dysmorphic features +/- major malformations +/- positive family history)	10	6	4	9%	3.6%

Baker et al [2002]	250	FISH	4 Reported	Highly selected (dysmorphic features +/- major malformations)	9	4	5*^d^*	3.6%	2%

Rio et al [2002]	150	Automated Fluorescent Genotyping	24% of the families studied	Highly selected (dysmorphic features +/- major malformations +/- positive family history)	12	9	3*^e^*	8%	2%

Van karnebeek et al [2002]	184	FISH	93 (positive family history of ID in the first, second or third degree relatives)	No selection	1	1	0	0.5%	0

Jalal et al [2003]	372	FISH	2 Reported	Selected (dysmorphic features)	23	15	8*^f^*	6.8%	2.15%%

Koolen et al [2004]	210	MLPA	2 Reported	No selection	9	7	2	4.3%	0.9%

Ravnan et al [2006]	11688	FISH	4 Reported	No selection	357	105/136 (136 parents studied)	31/136 (136 parents studied)	2.5%	0.7%

Rooms et al [2006]	275	MLPA	3 Reported	No selection	8	5	3	2.9%	1.1%

Ruiter et al [2007]	624	MLP	Not Reported	No selection		Not Reported	Not Reported	Not Reported	0.8%

Ahn et al [2007]	455	FISH, MLPA	Not Reported	No selection	27^g^	25*^g ^*	2*^h^*	5.9%*^g^*	0.4%

Stegmann et al [2008] *9*	466	MLPA	Not Reported	No selection	15	10	5	3.2%	1%

Ahn et al [2008] *i*	403	MLPA	Not Reported	No selection	17^*g*, j^	16*^g^*	1	5.5%*^g^*	0.2%

Shao et al [2008]	5380(patients with known and unknown Karyotype)	Array-CGH	Not Reported	No selection	236*^k^*	216*^k^*	20*^k^*	4.4%*^k^*	0.4%**
			
	2725 (patients with known Karyotype)		Not Reported	No selection	76	Not Reported	Not Reported	2.8%	Not Reported -

Wu et al [2010]	451	MLPA, SNP array	Not Reported	Selected (Moderate to severe ID)	23	19	4*^l^*	5.1%	0.9%

The present study	322	MLPA	102 (positive family history in the first degree relatives)	No selection	1	0	1	0.98%	0.98%

*^a ^*Two proved to be inherited and two were assumed to be inherited based on family history.

*^b ^*Degree of relationship not determined.

*^c ^*Five families confirmed, 1 not confirmed.

*^d ^*Mother normal, father not available (1 family).

^*e *^In one family with 4 affected siblings, the deletion was not detected in the parents, germline mosaicism or balanced translocation are suggested.

^*f *^Parents not available in 3 families.

*^g ^*polymorphisms are included.

*^h ^*Mother normal, father not available (1 family).

*^i ^*Abnormalities which were detected by Karyotype were excluded.

*^j ^*No information was available on the phenotype of the carrier parents (in 6 families).

*^k ^*Including abnormalities detected by Karyotype.

*^l ^*The details of the parental studies are not reported.

Based on the adopted inclusion criteria, theses investigations can be divided into two groups. The first group includes studies on highly selected "moderate to severe ID" patients with dysmorphic features and/or multiple congenital anomalies. Most of such early studies were carried out using FISH as the main method (Table 1). The overall frequency of subtelomeric changes ranged from 3.6% to 9% and the frequency of hereditary subtelomeric aberrations was 2%-3.6% [12,15-21].

Introduction of new molecular cytogenetic techniques such as MLPA and array-CGH in subtelomeric investigations, allowed the broad screening of ID patients. The second group of studies was taken place irrespective of the severity of intellectual disability or concurrent dysmorphism or congenital malformations [22-32]. In this group, the overall frequency of subtelomeric changes was 0.5-4.4% while that of the hereditary aberrations was 0-1.1%.

The main inclusion criteria of the present study was the recurrence of ID in the patients and their first degree relatives (positive family history) irrespective of the severity of ID or the presence of concurrent dysmorphism or congenital malformations. The frequency of subtelomeric rearrangements found here is in the range of hereditary subtelomeric aberrations of the second mentioned group. It can therefore be concluded that the positive family history, by alone, could not be considered as a significant indicator of subtelomeric aberrations in unselected ID patients, though it had previously been proposed as an important and determining factor [13,19,33].

Following the proposal of subtelomeric rearrangements as a significant cause of ID, Knight et al conducted the first large-scale study (466 ID patients) with a subtelomeric rearrangements frequency of 7.4% in moderate to severe ID patients and 0.5% in mild ID ones [11]. Almost half of the positive cases examined (10 out of 22), were hereditary unbalanced chromosomal translocations, among them, 9 families had positive family history. The authors concluded that selecting the patients with family history of ID could increase the ratio of informative tests from 7/100 in sporadic cases to 25/100 in familial moderate to severe ID cases leading to the reduction of the cost per informative tests.

Further studies also proposed the significant predictive value of the family history of ID in detecting subtelomeric aberrations. Riegel et al compared the prevalence of subtelomeric changes in two groups of patients with different inclusion criteria and concluded that the most important selection criterion was the presence of more than one affected individual in a family [19]. In 2001, De Vries et al compared clinical variables of 29 patients with known subtelomeric aberrations with 110 ID patients with unknown etiology and provided a five-item checklist to improve the diagnostic rate of subtelomeric defects [13]. Their results suggested prenatal onset growth retardation and positive family history for ID, as important indicators of subtelomeric rearrangements.

Subsequent studies were mostly carried out on sporadic ID cases. However, in several studies familial cases were also included. Among population studied by Rossi et al [16], Anderlid et al [12] and Rio et al [18], there were 53/200 (26.5%), 40/111 (36%) and 24% familial ID cases respectively. It is noteworthy that while in the mentioned investigations the term "positive family history" has been applied to the families with more than one affected individual in either their first, second or third degree relatives, the present study has been carried out only on patients and families in which there were more than two affected individuals in their first degree relatives.

Rosenberg et al reported the results of a study on 120 highly selected sporadic ID patients in whom the positive family history of ID had already been excluded [17]. Interestingly, they found 5 subtelomeric changes, among them 4 were hereditary unbalanced translocations. In accordance with the results of the present study, their findings show that where patients with moderate to severe ID, dysmorphism or congenital malformations are selected, the frequency of subtelomeric aberrations and the proportion of hereditary subtelomeric changes do not significantly decrease even if the positive family history is an exclusion criterion.

Van Karnebeek et al investigation included the highest number of familial cases (93 from 184 families, nearly 50% of the studied population) [31]. The detected prevalence of subtelomeric rearrangements was 0.5% (only one subtelomeric aberration found in a mild ID patient) which was lower than most of the reported frequencies. The results of our study could further explain their finding and the low detected prevalence could be attributed to the high proportion of familial cases.

## Conclusions

In conclusion, to the best of our knowledge, this study is the first investigation of a large sample set of familial ID cases to evaluate the contribution of subtelomeric rearrangements in familial ID which showed that it was not as much as what had been determined for sporadic ID in the literature. Our results propose that the positive family history by alone, cannot be the most important and determining indicator of subtelomeric aberrations. In other words, the presence of positive family history can increase the probability of subtelomeric changes in ID patients only if there are associated features demonstrating the severe forms of the disease like dysmorphism, multiple congenital abnormalities or moderate to severe ID. Moreover, our findings could explain the inconsistency of the results of some previous studies. More investigations including the assessment of copy number changes of the other genomic regions and the study of single gene disorders are therefore recommended to delineate the underlying genetic causes in the studied families.

## Methods

### Families and Patients

In this study, 102 families were selected based on the following criteria: 1) the presence of at least two affected individuals in the first degree relatives with ID (diagnosed according to the standard definition of ID) or multiple congenital anomalies (MCA) in case of neonates that development could not yet be evaluated 2) Normal karyotype of at least one of the affected individuals. 3) No Fragile-X expansion mutation found in at least one of the affected individuals 4) no evidence of metabolic, neurodegenerative or other single gene disorders based on the available previous investigations including brain imaging and blood/urinary metabolic screening. No additional clinical selection or classification according to the severity of the observed ID or the presence of the dysmorphic features was applied.

The study was approved by the Ethics Committee of Tehran University of Medical Sciences. The affected individuals, their parents and the normal siblings of each family, were all contacted to participate in this project. The families were informed about the objectives of this study to diagnosis and prevention of the ID recurrence and the details of the provided genetic tests in a genetic counseling session. Standard assessment included a record of medical history of prenatal, perinatal and postnatal period, admissions and medications used; an extended pedigree with special attention to the presence of ID, multiple congenital anomalies and recurrent abortion; and physical examination of ID patients. Relevant data were retrieved from the previous documented clinical and paraclinical investigations of the patients. An informed consent was obtained from all the participants or their guardians.

Pedigrees, medical information of family members including demographic data, physical examination and paraclinic findings and details of all genetic evaluations carried out on each individual sample were all entered in an integrated database.

Anticoagulated blood samples were collected from all family members including the parents, affected individuals and the normal siblings. Post-test genetic counseling was scheduled for all families.

### MLPA Analysis

Genomic DNA was extracted from anticoagulated peripheral blood samples according to the standard phenol-chloroform DNA extraction protocol. Subtelomeric rearrangements were studied using the Multiplex Ligation dependent Probe Amplification (MLPA) technique. The probemixes used, were SALSA MLPA kits P036 and P070 (MRC-Holland, Amsterdam, The Netherlands). These two probemixes are designed for evaluating the copy number differences of subtelomeric regions of all chromosomes except acrocentric arms of 13p, 14p, 15p, 21p and 22p, for which the probemixes include probes on the q arm, close to the centromere of these chromosomes.

Subtelomeric aberrations were studied using P036 probemixe in one of the affected individuals of each family. Detected rearrangements were then confirmed by P070 probemix, as the sequences detected by these two probemixes are specific for different genes in subtelomeric regions. Whenever a copy number variation was detected, other family members including the parents, other affected individuals and the normal siblings were then studied to differentiate the pathologic changes from familial variations. Pathologic changes detected by MLPA were then confirmed by fluorescence in situ hybridization (FISH) technique using appropriate subtelomeric probes.

Standard MLPA analysis was performed following the manufacturer's instructions. Briefly, 500 nanograms of genomic DNA was denatured and then hybridized with SALSA MLPA probes at 60°C for 16 hours. Following ligation at 54°C for 15 minutes, PCR was performed in a Gene Amp PCR system 9700 (Applied Biosystems, Foster City, CA, USA) using universal 6-FAM labeled primers supplied with the kit. Fluorescent amplification products were subsequently separated by capillary electrophoresis on an ABI 3130 Genetic Analyzer (Applied Biosystems, Foster City, CA, USA) and analysed using the Genemapper V4.0 software. DNA copy number was estimated using Coffalyser V9.4, which quantifies the ratio of peak areas in test samples over the peak areas of normal controls in each target sequence.

### FISH Analysis

Standard metaphase FISH analysis was carried out to confirm MLPA findings as previously described [34]. Briefly, metaphase chromosome spreads were prepared and spotted onto the cleaned microscope slides. Slides were immersed in 2x sodium saline citrate (SSC, pH 7) for 2 minutes and dehydrated in 70%, 85% and 100% ethanol series for 2 minutes each. Ten microliter of appropriate Cytocell subtelomeric specific probes (Cytocell, Ltd. Oxfordshire, UK) was applied onto the slides. After denaturing at 75°C for 2 minutes and overnight incubation at 37°C, post hybridization wash was performed and the slides were counterstained by DAPI. Microscopy was performed using a Leika DM 6000B (Leika Microsystems, Germany) fluorescent microscope equipped with a CCD camera and images were analyzed by Leika CW4000 software.

### Literature Review

Major investigations of subtelomeric rearrangements in ID patients are thoroughly reviewed with specific attention to the selection criteria, proportion of familial cases and differentiating the de novo abnormalities from the hereditary ones. The keywords used, were Familial Intellectual disability, Mental Retardation, Developmental Delay and Subtelomeric Rearrangements. The details of these investigations are summarized in a table.

To have a more precise estimation, just the studies with sample size of more than 100 patients were included in the review. Moreover, studies reported in non-English languages, or investigations carried out in recent years on limited number of patients using old techniques with no further new data provided, were not included [35,36].

## Competing interests

The authors declare that they have no competing interests.

## Authors' contributions

SRG designed and initiated the study, monitored data collection and analysis for the whole study and drafted and revised the paper. He is guarantor. MR contributed to the study design, implemented the technical parts, designed data collection tools, analyzed the data, and drafted and revised the paper. MRG initiated the collaborative project and designed the data collection tools. YH, HA, ZTK and JD helped with data collection and designed the data collection tools. MRE analyzed the data. AH and MP contributed to data collection and helped with technical parts. All authors have read and approved the final manuscript.
